# Ginsenoside Rb1 Enhances Atherosclerotic Plaque Stability by Improving Autophagy and Lipid Metabolism in Macrophage Foam Cells

**DOI:** 10.3389/fphar.2017.00727

**Published:** 2017-10-24

**Authors:** Lei Qiao, Xue Zhang, Minghao Liu, Xiaoling Liu, Mei Dong, Jing Cheng, Xinyu Zhang, Chungang Zhai, Yu Song, Huixia Lu, Wenqiang Chen

**Affiliations:** ^1^The Key Laboratory of Cardiovascular Remodeling and Function Research, Chinese Ministry of Education and Chinese Ministry of Health; the State and Shandong Province Joint Key Laboratory of Translational Cardiovascular Medicine, Department of Cardiology, Qilu Hospital of Shandong University, Jinan, China; ^2^Department of Cardiac Uhrasonography, Binzhou People’s Hospital, Binzhou, China

**Keywords:** ginsenoside Rb1, atherosclerosis, lipid accumulation, macrophage foam cells, autophagy

## Abstract

Atherosclerosis (AS) is a lipid-driven disease in which macrophage foam cells play a critical role by increasing vascular lipid accumulation and contributing to plaque instability. Ginsenoside Rb1 (Rb1), the most abundant active component of ginseng, has been found potently to promote lipid metabolism and attenuate lipid accumulation. However, the underlying mechanisms remain unclear. In this study, the effects of Rb1 on lipid accumulation and plaque stability were investigated both *in vitro* and *in vivo* by using primary peritoneal macrophages isolated from C57BL/6 mice and an AS model in ApoE^-/-^ mice. The results showed that Rb1 reduced lipid accumulation both in macrophage foam cells and atherosclerotic plaques. Rb1 treatment promoted plaque stability by modifying plaque composition via the activation of autophagy both *in vitro* and *in vivo.* Transmission electron microscopy further showed an increased accumulation of autophagolysosomes in Rb1-treated macrophage foam cells. However, the modulation of lipid accumulation by Rb1 was attenuated by autophagy blockage using autophagy-related gene 5 (Atg5) small interfering RNA (siRNA) *in vitro*. In addition, Rb1 notably increased AMPK phosphorylation both *in vitro* and *in vivo*, and the AMPK inhibitor compound C abolished the Rb1-induced autophagy in macrophage foam cells. In conclusion, ginsenoside Rb1 reduced lipid accumulation in macrophage foam cells and enhanced atherosclerotic plaque stability by the induction of macrophage autophagy. Our study provides new evidence for the possible use of Rb1 in the prevention and treatment of AS.

## Introduction

Atherosclerosis (AS) is a chronic lipid-driven disease in large and medium arterial walls (Weber and Noels, 2011). Macrophages serve as the major component of atherosclerotic lesions and are critical to the development of AS by engulfing ox-LDLs to form cholesterol-laden foam cells (Moore et al., 2013), contributing to the increase in atherosclerotic plaque complexity and instability. Therefore, decreasing lipid accumulation in foam cells and atherosclerotic plaque is an efficient way to alleviate or reverse the progression of AS.

Autophagy is an evolutionarily conserved process dedicated to the degradation of cytoplasmic materials, such as long-lived proteins and dysfunctional organelles (Levine and Kroemer, 2008). Previous studies have found that autophagy plays a vital role in lipid metabolism (Singh et al., 2009; Dong and Czaja, 2011), through facilitating the hydrolysis of intracellular lipids and promoting cholesterol efflux in macrophage foam cells (Ouimet et al., 2011). Impaired autophagy efflux of macrophages could lead to an ineffective whole-body clearance of accumulated lipids, which underlies the pathogenesis of many important metabolic disorders, including fatty liver, obesity and AS (Ouimet et al., 2011). Increasing evidence has demonstrated that dysfunctional autophagy is greatly associated with the progression of AS (Razani et al., 2012; Li et al., 2016). Incompetent autophagy is also responsible for excessive lipid accumulation in atherosclerotic plaques. Thus, therapeutic strategies to increase autophagic activity may provide a new approach to facilitate lipid metabolism and plaque stability in advanced atherosclerotic plaques.

Ginseng, a traditional herbal medicine, has been widely used in Eastern Asia and the western world for its alleged tonic effect and restorative properties. Previous studies have shown that ginsenoside Rb1, the most abundant active component of ginseng (Washida and Kitanaka, 2003), plays a protective role in many cardiovascular diseases. For instance, Rb1 prevents homocysteine-induced endothelial dysfunction (Lan et al., 2011), and protects against cardiac remodeling (Zheng et al., 2017) and ischemia/reperfusion-induced myocardial injury (Cui et al., 2017). However, the role of Rb1 in AS has not been reported. Considering the ability of Rb1 to promote lipid metabolism and attenuate lipid accumulation (Park et al., 2002; Shen et al., 2013; Yu et al., 2015), we hypothesized that Rb1 may promote atherosclerotic plaque stability through facilitating lipid metabolism in macrophage foam cells.

To test this hypothesis, primary peritoneal macrophages isolated from C57BL/6 mice and an AS model in the ApoE^-/-^ mouse background were used to explore whether ginsenoside Rb1 was cardioprotective by mitigating lipid accumulation in macrophage foam cells and investigate its mechanism.

## Materials and Methods

### Ethics Statement

All animal experimental protocols complied with the Animal Management Rules of the Chinese Ministry of Health (document no. 55, 2001) and conformed to the NIH guidelines (the Guide for the Care and Use of Laboratory Animals published by the National Institutes of Health; NIH Publication No. 85-23, revised 1996).

### Cell Culture and Treatment

Mouse primary peritoneal macrophages were obtained as described (Ho et al., 2012). Briefly, 3% thioglycollate was intraperitoneally injected into mice. After 3 days, mice were first anesthetized with a lethal dose of isoflurane and then euthanized by cervical dislocation. Peritoneal macrophages were collected by peritoneal lavage using cold PBS. The cell pellets were obtained after centrifugation at 800 rpm for 5 min. The cells were incubated in 1640 RPMI supplemented with 10% FBS and 1% antibiotics for 3 h and washed three times to remove non-adherent cells. Cells were incubated with ox-LDL (100 μg/ml, Peking Union-Biology, China) for 24 h to induce macrophage foam cell formation. Then, cells were treated with different concentrations of ginsenoside Rb1 (Fleton Natural Products Co. Ltd.) or PBS for another 24 h. Cells were also treated with compound C (CC, 20 μM, sc200689, Santa Cruz Biotechnology, United States) for 2 h following the addition of Rb1 for 24 h. Macrophage foam cells were stained with 0.5% oil red O.

### Oil Red O Staining

Cultured primary peritoneal macrophages were plated on cover slides in six-well plates and incubated with ox-LDLs for 24 h to form macrophage foam cells and different concentrations of ginsenoside Rb1 or PBS for another 24 h. Cells were washed with cold phosphate-buffered saline (PBS) three times and fixed with 4% paraformaldehyde for 30 min. Subsequently, 0.5% oil red O was added to cells for 30 min. Of note, oil red O needs to be filtered to remove impurities. Foam cells were observed under a microscope at ×100 and ×200 magnification.

### Western Blotting

Protein extraction and western blotting were performed as described before (Ho et al., 2009). Peritoneal macrophages were washed with ice-cold PBS and lysed on ice using RIPA lysis buffer supplemented with complete protease inhibitor cocktail. Equal amounts of protein (20 μg) were separated by 12% SDS-PAGE gel electrophoresis and transferred onto PVDF membranes (Bio-Rad). Membranes were blocked in 5% non-fat dry milk followed by incubation with primary antibodies. Antibodies purchased from Abcam (Cambridge, United Kingdom) were as follows: LC3B (ab48394), SQSTM1/p62 (ab56416) and Atg5 (ab108327). Antibodies obtained from Cell Signaling Technology (Danvers, MA, United States) included those against AMPKα (5831) and p-AMPKα (Thr172) (2535). Secondary antibodies (ZSJB-BIO, China) were horseradish peroxidase (HRP)-conjugated goat anti-rabbit or goat anti-mouse antibodies.

### Transfection of siRNA

Small interfering RNA (Biology Engineering Corporation, Shanghai, China) was used to block the expression of Atg5. The mRNA sequence of the Atg5 RNAi was 5′-ACCGGAAACTCATGGAATA-3′ (Seo et al., 2011). Mouse primary peritoneal macrophages (1 × 10^6^ cells/ml) were transfected with con siRNA or Atg5 siRNA using Lipofectamine 2000. After 24 h, western blotting was performed to confirm the knockdown of Atg5 protein for each experiment.

### Transmission Electron Microscopy (TEM)

Cells were fixed with 4% glutaraldehyde prior to postfixation in 1% osmium tetroxide. Samples were processed and embedded in Epon-Araldite resin. Ultrathin sections were obtained by using an ultramicrotome and counterstained with lead citrate. A Hitachi H-7500 electron microscope was used to observe lipid droplets and autophagosomes.

### Animals

A total of 30 apoE^-/-^ mice (male, 8 weeks old) were obtained from Charles River Laboratories (Beijing, China), and all mice were kept on a 12-h light/12-h dark cycle. All mice were fed a high-fat diet (0.25% cholesterol and 15% cocoa butter) for 20 weeks. Subsequently, ApoE^-/-^ mice were randomly divided into 2 groups (*n* = 15 per group) for treatment: the Rb1 group received an intraperitoneal injection with Rb1 (50 mg/kg/d) for 8 weeks and the control group, an equal volume of saline. After 8 weeks of treatment, mice were sacrificed, and the aorta and heart were harvested. Blood samples were collected to measure serum lipid levels.

### Histopathological Staining and Immunofluorescence Analysis

Mice were sacrificed, and the heart with attached aortic roots was obtained immediately and fixed overnight in 4% formaldehyde. The aortic roots were embedded in optimum cutting temperature (OCT) compound for cryosections, and were cut into serial 6-μm cross-sections for histological staining and immunofluorescence. IHC staining was undertaken using the rabbit monoclonal antibody against smooth muscle actin (ab5694; Abcam, United States) and rat monoclonal antibody against MOMA-2 (MCA519G; AbD, United Kingdom). The sections were incubated with horseradish peroxidase (HRP)-conjugated goat anti-rabbit or goat anti-rat secondary antibodies (ZSJB-BIO, China). The lipid and collagen content of lesions was detected by oil-red O staining and sirius red staining, respectively. The slides were quantitatively analyzed using Image-Pro Plus 6.0 (IPP 6.0, Media Cybernetics, Rockville, MD, United States).

### Statistical Analysis

All values are representative of at least three independent experiments and expressed as the means ± SEM by use of GraphPad Prism 5 (La Jolla, CA, United States). Differences among groups were analyzed by one-way ANOVA followed by a Tukey *post hoc* test, and *t*-test was used for the two-group comparisons. Statistical significance was defined as *P* < 0.05.

## Results

### Rb1 Reduced Lipid Accumulation in Macrophage Foam Cells

**FIGURE 1:**
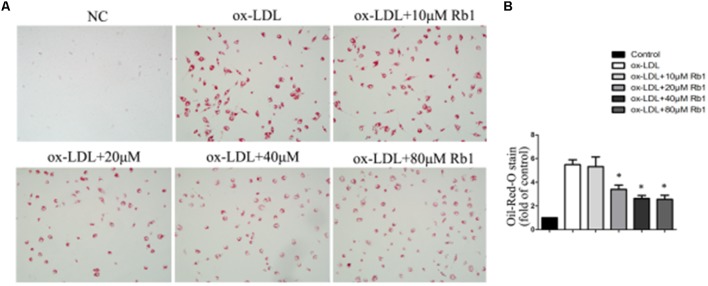
Rb1 decreased lipid accumulation in macrophage foam cells. Primary peritoneal macrophages from C57BL/6 mice were incubated with ox-LDL (100 μg/ml) for 24 h and further incubated in the absence or presence of different doses of Rb1 for another 24 h. **(A,B)** oil red O staining was performed to assess lipid accumulation in macrophages.^∗^*p* < 0.05, compared to the ox-LDL group, *n* = 3.

Rb1 has been reported to be able to reduce liver lipid accumulation (Shen et al., 2013; Yu et al., 2015). Here, we investigated whether Rb1 had an effect on intracellular lipid accumulation in macrophage foam cells. Macrophages were exposed to 100 μg/ml ox-LDLs for 24 h and further incubated in the absence or presence of 10–80 μM Rb1. Oil red O was used to analyze intracellular lipid accumulation. Treatment of macrophages with ox-LDLs for 24 h resulted in foam cell formation which was characterized by heavy lipid loading. Incubation with Rb1 for another 24 h reduced lipid accumulation, with the minimum effect found at a dose of 20 μM (**Figures 1A,B**). Therefore, Rb1 reduced lipid accumulation of macrophage foam cells *in vitro.*

### Rb1 Rescued the Impaired Autophagy Flux in Macrophage Foam Cells

**FIGURE 2:**
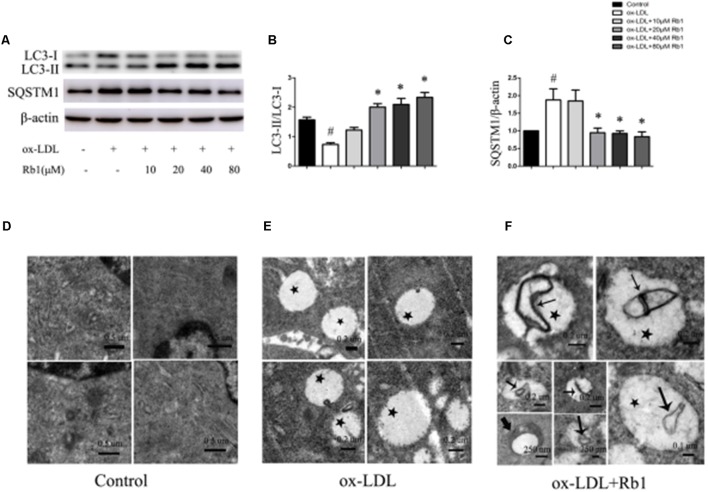
Rb1 rescued the impaired autophagy flux in macrophage foam cells. **(A)** Representative western blot analysis of LC3B and SQSTM1/p62. **(B)** Quantification of LC3 and **(C)** SQSTM1/p62 expression relative to the β-actin level in different groups. Electron microscopic analysis of autophagosomes in macrophage foam cells treated with Rb1. Primary peritoneal macrophages were incubated with **(D)** PBS or **(E)** ox-LDL (100 μg/ml) for 24 h, and further incubated with **(F)** Rb1 (20 μM ) for another 24 h. Asterisks indicate lipid droplets (LDs); arrows indicate double-membrane vesicles analogous to autophagosomes (APs) in or around LDs. ^∗^*p* < 0.05, compared to the ox-LDL group; ^#^*p* < 0.05, compared to the control group, *n* = 3.

Autophagy has been found to play critical roles in lipid metabolism (Singh et al., 2009; Dong and Czaja, 2011; Ouimet et al., 2011). The LC3II/LC3I ratio and SQSTM1/p62 protein have been considered to be a standard markers for the detection of autophagy (Mizushima and Yoshimori, 2007; Klionsky et al., 2012). The results showed that treatment with ox-LDLs for 24 h decreased the LC3II/LC3I ratio (**Figures 2A,B**) with a corresponding increase in the SQSTM1/p62 level in peritoneal macrophages (**Figures 2A–C**), suggesting that autophagy flux was impaired in cholesterol-laden foam cells. The result was consistent with the previous reports (Singh et al., 2009; Koga et al., 2010; Gu et al., 2016) which showed that increased lipid content might impair autophagy flux. Interestingly, Rb1 treatment rescued the autophagy flux impaired by ox-LDLs in macrophage foam cells, which was evidenced by the increased LC3II/LC3I ratio and SQSTM1/p62 degradation (**Figures 2A–C**).

To further examine the effect of Rb1 on autophagy in macrophage foam cells, transmission electron microscopy (TEM) was used to explore morphological evidence for autophagy flux. Little lipid droplets (LDs) or autophagosomes (APs) were observed in primary peritoneal macrophages with no treatment (**Figure 2D**). Treatment with ox-LDL caused lipid accumulation in the form of LDs, which were easily identifiable as round light-density structures without a bilayer lipid membrane (**Figure 2E**). Rb1 treatment increased the number of double-membrane autophagosomes (APs), which were presented in or around LDs (**Figure 2F**), suggesting that autophagy occurred on the surface of the LDs. Taken together, our data suggested that Rb1 rescued the impaired autophagy flux in macrophage foam cells.

### Rb1 Reduced Lipid Accumulation of Macrophage Foam Cells by Autophagy Induction

**FIGURE 3:**
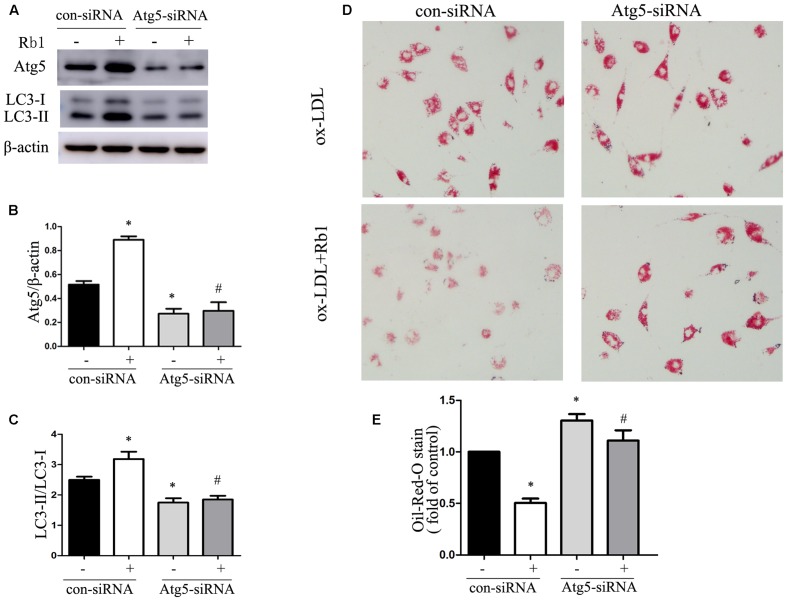
Rb1 reduced lipid accumulation of macrophage foam cells by autophagy induction. Primary peritoneal macrophages were transfected with con siRNA or Atg5 siRNA and then treated with ox-LDL (100 μg/ml) in the absence or presence of 20 μM Rb1. **(A)** Representative western blot analysis of Atg5 and LC3. **(B)** Quantification of Atg5 and **(C)** LC3 expression relative to β-actin in macrophages with different treatments. **(D)** Lipid accumulation in macrophages transfected with Atg5 siRNA with or without Rb1 treatment. **(E)** Quantitative analysis of lipid accumulation. ^∗^*p* < 0.05, compared to con siRNA; ^#^*p* < 0.05, compared to con siRNA + Rb1, *n* = 3.

Because of the demonstrated reduction in lipid accumulation in macrophage foam cells, accompanied by increased expression of autophagy proteins and autophagosomes after Rb1 treatment, we speculated that Rb1 treatment may decrease lipid accumulation by activating autophagy flux in macrophage foam cells. To test this hypothesis, autophagy-related gene 5 (Atg5) small interfering RNA (siRNA) was applied to block autophagic flux in macrophages. Macrophages transfected with Atg5 siRNA showed a decreased level of Atg5 and LC3 II expression (**Supplementary Figure S1**). In the control siRNA group, Rb1 (20 μM) increased the Atg5 expression and LC3II/LC3I ratio significantly (**Figures 3A–C**) and reduced the lipid droplet accumulation indicated by oil red O staining (**Figures 3D,E**). In contrast, in macrophages transfected with Atg5 siRNA, Rb1 failed to alter the levels of autophagy proteins or attenuate intracellular lipid accumulation. These data suggested that Rb1 reduced lipid accumulation of macrophage foam cells by autophagy induction.

Previous studies have shown that autophagy regulates cholesterol efflux from macrophage foam cells, in which ATP-binding cassette transporter A1 (ABCA1) plays a vital role (Ouimet et al., 2011). As we have proven that Rb1 could promote lipid metabolism in macrophage foam cells via autophagy induction, we indirectly evaluated cholesterol efflux by detecting the expression of ABCA1. Our results showed a significant increase in ABCA1 in macrophage foam cells upon Rb1 treatment, which provides indirect evidence for cholesterol efflux (**Supplementary Figure S2**).

### Rb1 Enhanced Atherosclerotic Plaque Stability by Modifying the Plaque Components

**FIGURE 4:**
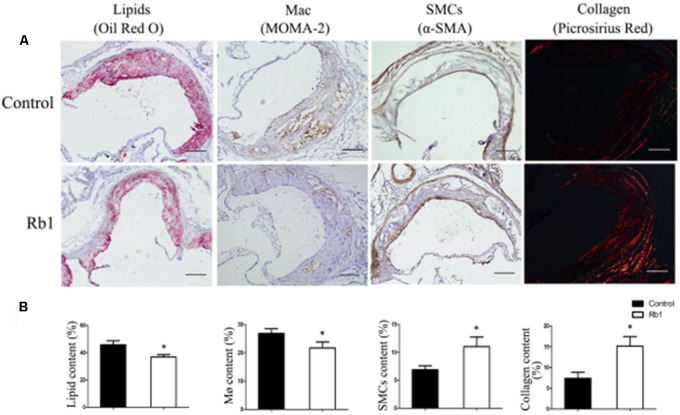
Effect of Rb1 treatment on plaque stability of apoE^-/-^ mice. **(A)** Immunohistochemical staining of oil red O staining (lipids), MOMA-2 (macrophages), α-SMA (smooth muscle cells) and sirius red staining for the quantification of collagen. **(B)** Area measurement of plaque components. Scale bar: 100 μm. ^∗^*p* < 0.05, compared to the control group, *n* = 6.

The results from our research (**Figures 1A,B**) as well as previous studies (Shen et al., 2013; Yu et al., 2015) showed that Rb1 contributed to reducing lipid accumulation. We next investigated the role of Rb1 in lipid accumulation *in vivo* in atherosclerotic plaque. Oil red O staining showed that the intraplaque lipid content was significantly decreased in the Rb1 treatment groups, along with decreased macrophage infiltration by histopathological staining (**Figures 4A,B**). This result was consistent with the *in vitro* results described above (**Figures 1A,B**). However, there was no significant difference in serum lipid profiles or blood glucose between the two groups of ApoE^-/-^ mice (Supplementary Table 1). Recent studies have shown that ginsenoside Rb1 plays a protective role in many cardiovascular diseases (Lan et al., 2011; Cui et al., 2017; Zheng et al., 2017); we subsequently investigated the effect of Rb1 on plaque stability. Unstable plaques are characterized by extensive macrophage infiltration and lipid accumulation, as well as a thin cap with less collagen and smooth muscle cell (SMCs) (Dong et al., 2013; Wang et al., 2014). Therefore, the intraplaque content of SMCs and collagen was additionally analyzed by sirius red staining and histopathological staining. The results showed that both of these two beneficial components were markedly increased in the Rb1 treatment groups (**Figures 4A,B**). Taken together, our data suggested that Rb1 enhanced atherosclerotic plaque stability by modifying the plaque components.

### Rb1 Increased Autophagy Levels in Atherosclerotic Plaques

**FIGURE 5:**
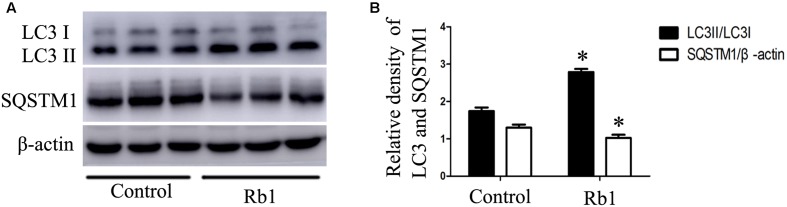
Rb1 increased autophagy levels in atherosclerotic plaques in apoE^-/-^ mice. **(A)** Representative western blot analysis and **(B)** quantification of LC3-II and SQSTM1/p62 *in vivo*. ^∗^*p* < 0.05, compared to the control group, *n* = 6.

Macrophage autophagy plays a protective role in AS (Liao et al., 2012). Advanced AS is accompanied by impaired autophagy flux with decreased Atg5 protein (Li et al., 2016) and increased SQSTM1/p62 protein (Razani et al., 2012). To investigate the role of Rb1 in autophagy activity in atherosclerotic plaques, we prepared aortic lysates from ApoE^-/-^ mice to analyze the protein levels of LC3II and SQSTM1/p62. Not surprisingly, Rb1 treatment promoted the expression of LC3II protein and SQSTM1/p62 degradation in these lysates (**Figures 5A,B**). These results indicated that Rb1 treatment increased autophagy levels in atherosclerotic plaques, which was possibly responsible for the decrease in the intraplaque lipids content and cardioprotective benefits induced by Rb1.

### Rb1 Induced Autophagy via Promotion of AMPK Phosphorylation

**FIGURE 6:**
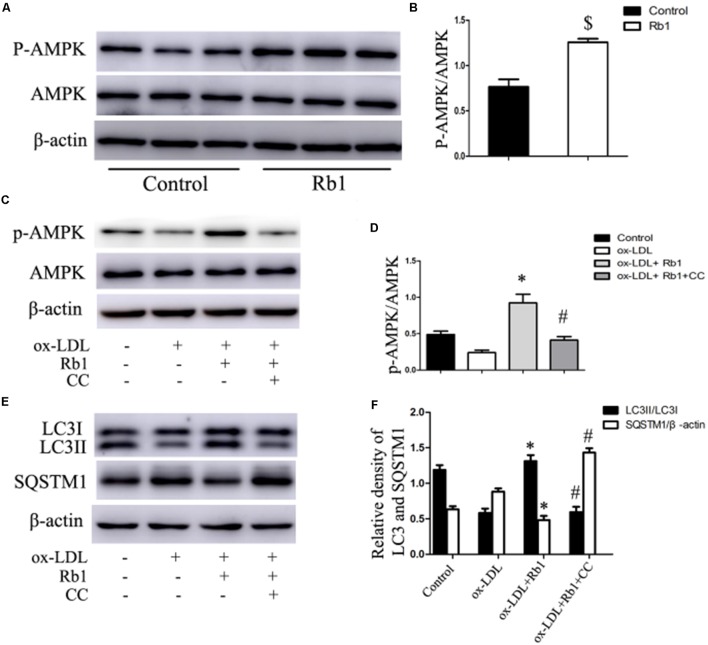
Rb1 induced autophagy via promotion of AMPK phosphorylation. **(A)** Representative western blots and **(B)** quantification of p-AMPK/AMPK in atherosclerotic plaques of apoE^-/-^ mice treated with Rb1 or saline (control). **(C)** Representative western blots and **(D)** quantification of p-AMPK/AMPK in macrophage foam cells treated with Rb1 in the presence or absence of 20 μM compound C (CC). **(E)** Representative western blots and **(F)** quantification of LC3-II and SQSTM1/p62 in macrophage foam cells treated with Rb1 in the presence or absence of 20 μM CC. ^∗^*p* < 0.05, compared to the ox-LDL group; ^#^*p* < 0.05, compared to the ox-LDL + Rb1 group; ^$^*p* < 0.05, compared to the control group, *n* = 6.

Previous studies have reported that Rb1 could promote AMPK phosphorylation (Shen et al., 2013; Shen et al., 2015). Therefore, the involvement of AMPK in Rb1-induced autophagy was evaluated in our study. The results showed that Rb1 treatment promoted AMPK phosphorylation both *in vivo* in atherosclerotic plaques (**Figures 6A,B**) and in macrophage foam cells (**Figures 6C,D**). Moreover, compound C, an AMPK inhibitor, abolished the increase in LC3II and SQSTM1/p62 degradation induced by Rb1 *in vitro* (**Figures 6E,F**). These data indicated that AMPK played a key role in Rb1-induced autophagy in macrophage foam cells.

## Discussion

Ginseng is widely used as a general tonic, adaptogen and herbal medicine for many diseases (Dong et al., 2013). Pharmacological effects of ginseng and its constituents have been demonstrated in the CNS and in the endocrine, cardiovascular, and immune systems (Attele et al., 1999). Most pharmacological properties of ginseng are attributed to ginsenosides (Attele et al., 1999). More than 20 ginsenosides have been isolated so far (Gillis, 1997). Ginsenosides exhibit considerable structural variation. Based on chemical structure, there are two major groups: panaxadiols (for example, Rb1, Rb2, Rg3, Rh2 and Rh3) and panaxatriols (for example, Re, Rg1, Rg2 and Rh1) (Attele et al., 1999). Ginsenosides belong to a family of steroids named steroidal saponins, which share structural features with steroid hormones (Kim et al., 1998). Ginsenosides exhibit diverse pharmacological effects. Rb1, Rg1 and Re have been shown to facilitate learning and memory (Takemoto et al., 1984; Salim et al., 1997); Rh2 and Rg3 have been reported to have antineoplastic and immunomodulatory effects (Tode et al., 1993; Mochizuki et al., 1995). Among these ginsenosides, Rb1 is the most abundant one. Rb1 has the structure of a tetracyclic triterpenoid with the molecular formula C54H92O23 and molecular weight of 1,109.26 g/mol (Cho et al., 2004; Xiong et al., 2010). Rb1 shows a wide range of biological activities. For example, Rb1 has a great potential for exerting anti-obesity effects by stimulating c-Fos expression in brain areas involved in energy homeostasis (Xiong et al., 2010); Rb1 also promotes glucose homeostasis by increasing insulin sensitivity (Shen et al., 2015). Recent studies have shown that Rb1 is cardioprotective through endothelial protection (Lan et al., 2011), anti-remodeling of ventricular (Zheng et al., 2017) and protection against IH-induced myocardial injury (Cui et al., 2017). However, the function of Rb1 in AS has not been elucidated.

Atherosclerosis is a chronic lipid-driven disease in the arterial wall. Macrophage foam cells play a crucial role in the development of atherogenesis. Excessive lipid deposition in the arterial wall leads to unbalanced cell apoptosis and inflammatory responses, which contributes to robust increases in atherosclerotic plaque complexity. Thus, removing excess cholesterol from macrophage foam cells in atherosclerotic plaques is necessary. In this study, we observed a reduction in lipid accumulation both in macrophage foam cells and in atherosclerotic plaques upon Rb1 treatment. In addition, Rb1 enhanced atherosclerotic plaque stability by modifying the plaque components to a more stable type. These results showed that Rb1 had potential therapeutic value in AS by decreasing lipid accumulation in macrophage foam cells.

Growing evidence has shown that macrophage autophagy plays a vital role in lipid metabolism and in AS (Singh et al., 2009; Dong and Czaja, 2011; Liao et al., 2012). Autophagy contributes to the hydrolysis of LDs and promotes cholesterol efflux in macrophage foam cells (Dong and Czaja, 2011; Ouimet et al., 2011). Autophagy is active in early atherosclerotic lesions and may alleviate foam cell formation. Autophagic clearance of lipids should be more efficient with increased lipid deposition in advanced atherosclerotic plaques. However, autophagy is increasingly dysfunctional in the progression of AS (Razani et al., 2012; Li et al., 2016). Impaired autophagy efflux results in ineffective clearance of accumulated cholesterol from foam cells. Therefore, pharmacological induction of autophagy in advanced atherosclerotic plaques might be a promising therapeutic approach to promote lipid metabolism and inhibit necrotic core formation.

To investigate whether autophagy was involved in the modulation of lipid content caused by Rb1, western blot and TEM were applied. Our results showed that Rb1 triggered autophagy by increasing the levels of LC3II and the degradation of SQSTM1/p62 and contributed to accumulated number of autophagosomes in macrophage foam cells *in vivo* and atherosclerotic plaque. Nevertheless, the effect of Rb1 on lipid accumulation was attenuated when autophagy was blocked by using Atg5 siRNA *in vitro*. Thus, our research showed for the first time that Rb1 reduced lipid accumulation in macrophage foam cells and enhanced atherosclerotic plaque stability by induction of macrophage autophagy.

**FIGURE 7:**
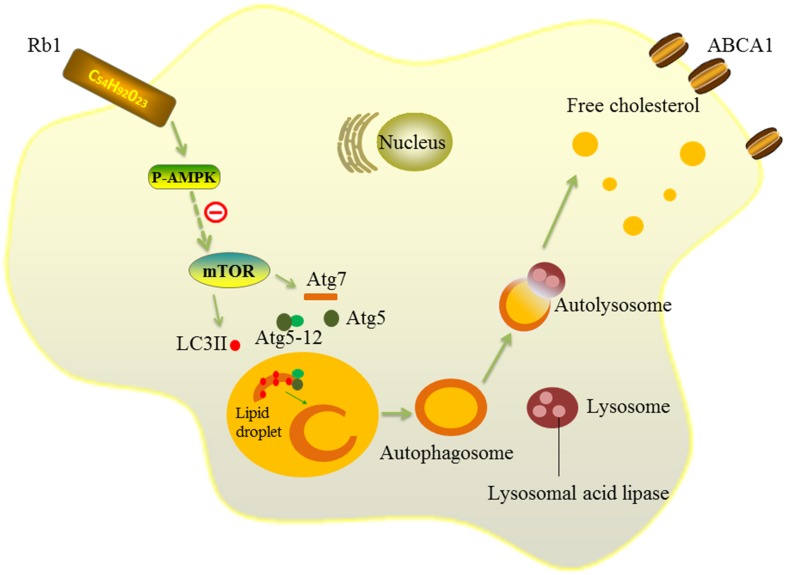
Schematic diagram of the effects of Rb1 on autophagy. Rb1 increased AMPK phosphorylation and thus induced autophagy activation in macrophage foam cells. Lipid droplets (LDs) are separated by double-membrane vesicles analogous to autophagosomes and delivered to lysosomes to form an autolysosome. LDs are hydrolyzed by lysosomal acid lipase (LAL) into free cholesterols in the autolysosome. Solid lines ( ) depict the mechanism by which Rb1 improves autophagy and lipid metabolism in macrophage foam cells in our present study. Dotted lines( ) represent a possible signaling molecule (mTOR) that may serve as an upstream regulator of autophagy induced by Rb1, but was not investigated in our study.

Then, we further explored the mechanism by which Rb1 restores autophagy flux in macrophage foam cells. AMPK is an evolutionarily conserved sensor of cellular energy receptor and plays a critical role in glucose and lipid metabolism, as well as the induction of autophagy (Kahn et al., 2005; Zhang et al., 2016). Our previous studies have shown that a lower AMPK activation level was associated with coronary atherosclerotic plaque vulnerability by attenuating autophagy in peripheral blood monocytes (Cheng et al., 2015). In addition, Rb1 has also been shown to be able to promote AMPK phosphorylation in rats (Shen et al., 2013, 2015). Therefore, AMPK was studied in our research to explore the potential molecular mechanisms by which Rb1 induced autophagy. The results showed that Rb1 treatment promoted AMPK phosphorylation both *in vitro* and *in vivo* in atherosclerotic plaques; the increase in LC3II and SQSTM1/p62 degradation induced by Rb1 was abolished by the AMPK inhibitor compound C *in vitro*. These data suggested that Rb1 induced autophagy via promotion of AMPK phosphorylation in macrophage foam cells (**Figure 7**).

## Conclusion

All of our findings suggest that ginsenoside Rb1 enhances atherosclerotic plaque stability by improving autophagy and lipid metabolism in macrophage foam cells. Our study supports the possibility that Rb1 might be an optional anti-atherogenic medicine by inducing macrophage autophagy in advanced atherosclerotic plaques.

## Author Contributions

HL, WC, and LQ conceived and designed experiments; and XZ, ML, XL, and MD conducted the experiments; JC and XZ analyzed the data; CZ and YS made the figures for this manuscript.

## Conflict of Interest Statement

The authors declare that the research was conducted in the absence of any commercial or financial relationships that could be construed as a potential conflict of interest. The reviewer CY and handling Editor declared their shared affiliation.

## Supplementary Material

**FIGURE S1 |** Autophagy was impaired in macrophages by using Atg5 small interfering RNA (siRNA). **(A)** Representative western blots and **(B)** quantification of Atg5/β-actin and LC3 II/β-actin in macrophages transfected with con siRNA or Agt5 siRNA. ^∗^*p* < 0.05, compared to the con siRNA group, *n* = 3.

**FIGURE S2 |** Rb1 treatment promoted the expression of ABCA1 in macrophage foam cell. **(A)** Representative western blots and **(B)** quantification of ABCA1/β-actin in macrophage foam cells treated in the presence or absence of 20 μM Rb1. ^∗^*p* < 0.05, compared to the control group; #*p* < 0.05, compared to the ox-LDL group, *n* = 3.
