# Corrigendum: Ginsenoside Rb1 Enhances Atherosclerotic Plaque Stability by Improving Autophagy and Lipid Metabolism in Macrophage Foam Cells

**DOI:** 10.3389/fphar.2017.00964

**Published:** 2018-01-09

**Authors:** Lei Qiao, Xue Zhang, Minghao Liu, Xiaoling Liu, Mei Dong, Jing Cheng, Xinyu Zhang, Chungang Zhai, Yu Song, Huixia Lu, Wenqiang Chen

**Affiliations:** ^1^The Key Laboratory of Cardiovascular Remodeling and Function Research, Chinese Ministry of Education and Chinese Ministry of Health; the State and Shandong Province Joint Key Laboratory of Translational Cardiovascular Medicine, Department of Cardiology, Qilu Hospital of Shandong University, Jinan, China; ^2^Department of Cardiac Uhrasonography, Binzhou People's Hospital, Binzhou, China

**Keywords:** ginsenoside Rb1, atherosclerosis, lipid accumulation, macrophage foam cells, autophagy

In the published article, there was an error in affiliation [1 and 2]. Instead of “^1^The Key Laboratory of Cardiovascular Remodeling and Function Research, Chinese Ministry of Education and Chinese Ministry of Health, Jinan, China, ^2^The State and Shandong Province Joint Key Laboratory of Translational Cardiovascular Medicine, Department of Cardiology, Qilu Hospital, Shandong University, Jinan, Shandong, China”, it should be “^1^The Key Laboratory of Cardiovascular Remodeling and Function Research, Chinese Ministry of Education and Chinese Ministry of Health; the State and Shandong Province Joint Key Laboratory of Translational Cardiovascular Medicine, Department of Cardiology, Qilu Hospital of Shandong University, Jinan, Shandong, China.” The authors apologize for this error and state that this does not change the scientific conclusions of the article in any way.

The original article has been updated.

## Conflict of interest statement

The authors declare that the research was conducted in the absence of any commercial or financial relationships that could be construed as a potential conflict of interest.

