# Supplementary figures and images for: Ginsenoside Rb1 Enhances Atherosclerotic Plaque Stability by Improving Autophagy and Lipid Metabolism in Macrophage Foam Cells
Source: Front Pharmacol. 2017 Oct 24;8:727. doi: 10.3389/fphar.2017.00727 (PMC5660703; doi:10.3389/fphar.2017.00727)

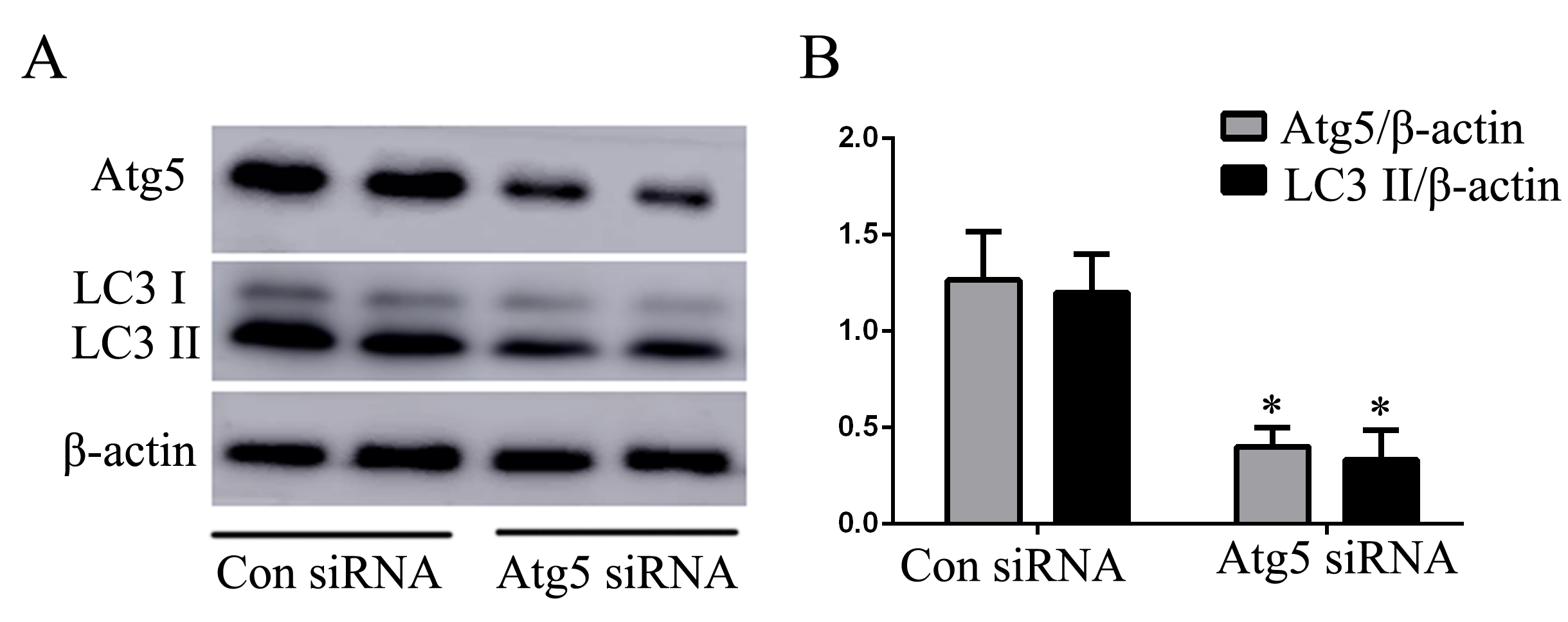

Supplement: Supplementary file 1 [file Image_1.TIF]

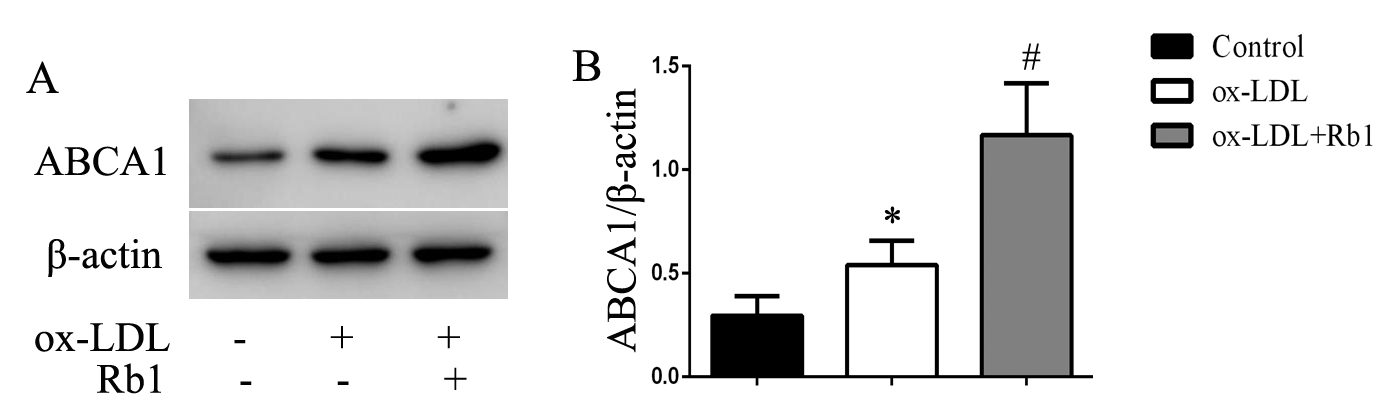

Supplement: Supplementary file 2 [file Image_2.TIF]
