# Supplementary material for: Ginsenoside Rb1 Enhances Atherosclerotic Plaque Stability by Improving Autophagy and Lipid Metabolism in Macrophage Foam Cells
Source: Front Pharmacol. 2017 Oct 24;8:727. doi: 10.3389/fphar.2017.00727 (PMC5660703; doi:10.3389/fphar.2017.00727)
Supplement: Supplementary file 3 [file Table_1.DOC]

Table 1: Effect of Rb1 on serum lipid profiles and blood glucose of apoE^-/-^ mice.

| **Groups TC TG HDL-C LDL-C glucose**  **(mmol/L) (mmol/L) (mmol/L) (mmol/L) (mmol/L)** |
| --- |
| Control 20.12±0.92 2.74±0.20 6.40±0.18 4.01±0.35 8.50±0.54  Rb1 18.12±0.16 3.10±0.10 7.12±0.43 3.55±1.47 8.17±0.61  *P* ns ns ns ns ns |

Data are expressed as mean±SEM; TC: total cholesterol; TG: triglyceride; HDL-C: high-density lipoprotein; LDL-C: low-density lipoprotein; ns: not significant; n=13
